# Gestational benzene exposure programs sex-specific neuroimmune responses to postnatal viral infection

**DOI:** 10.3389/fimmu.2026.1952071

**Published:** 2026-09-30

**Authors:** Anthony Maxwell, Annie Thy Nguyen, DruAnne Maxwell, Savannah Schick, Apoorva Rao, Aditi Singh, Elizabeth Findeis, Jiahui Ding, Gil Mor

**Affiliations:** 1 C.S. Mott Center for Human Growth and Development, School of Medicine, Wayne State University, Detroit, MI, United States; 2 Department of Biochemistry, Microbiology and Immunology, School of Medicine, Wayne State University, Detroit, MI, United States; 3 Department of Obstetrics and Gynecology, School of Medicine, Wayne State University, Detroit, MI, United States

**Keywords:** benzene, fetal brain, GPCR signaling, microglia, neuroimmune programming, placenta-brain axis, prenatal exposure, viral infection

## Abstract

**Background:**

Benzene is a widespread volatile organic compound, but its effects on fetal neuroimmune development remain poorly defined. We tested whether continuous gestational benzene exposure alters fetal placenta–brain signaling, fetal brain myeloid phenotype, and selected postnatal responses to viral challenge.

**Methods:**

Pregnant C57BL/6 mice were exposed to filtered air or 5 ppm benzene continuously from E0.5 to E12.5 or E17.5. Fetal tissues were collected at the respective endpoints. A separate cohort exposed through E17.5 delivered naturally; offspring received MHV68 at PND10 and were analyzed at PND17. We assessed fetal morphometry, sex-stratified transcriptomic and qPCR profiles, and brain myeloid-cell phenotypes.

**Results:**

Benzene exposure was associated with a female-specific reduction in occipitofrontal diameter at E12.5 that was not detected at E17.5. Transcriptomic and qPCR analyses identified sex-dependent differences at the two fetal collection endpoints in developmental, immune, and GPCR-linked signaling pathways. At E17.5, benzene exposure reduced EGR2 and increased MHC II in fetal brain myeloid/microglia-enriched cells in both sexes. At PND17 after MHV68 challenge, prenatally exposed males had higher brain ORF65 and *Il1b* transcript abundance and altered IBA1/IL-1β responses; no corresponding benzene-associated differences were detected among the female endpoints measured.

**Conclusions:**

Continuous gestational benzene exposure was associated with sex-dependent alterations in fetal neuroimmune measures and selected postnatal responses to viral challenge. Because the E12.5 and E17.5 cohorts differed in developmental age, cumulative exposure duration, and neural tissue collected (fetal head versus fetal brain), the study does not identify a discrete gestational window of susceptibility.

## 1 Introduction

Benzene is a widespread volatile organic compound encountered through gasoline, vehicle exhaust, tobacco smoke, combustion sources, industrial emissions, and indoor environments, including residences and workplaces (1–3). Although benzene is best known as a hematotoxicant and established human carcinogen, pregnancy represents a distinct period of developmental vulnerability (4). During gestation, environmental exposures can disrupt placenta–brain communication whereas fetal detoxification and immune-response pathways are still developing (5–8). Our prior work showed that fetal sex modifies the placental response to gestational benzene exposure (9), and experimental studies have linked prenatal benzene exposure with altered offspring hypothalamic development and later metabolic phenotypes (8). Epidemiologic studies have associated prenatal benzene exposure with adverse birth outcomes, including altered fetal growth and head circumference, whereas experimental work suggests effects on placental inflammation, fetal development, and offspring immune responses (10–12). Together, these observations provide a rationale to determine whether gestational benzene exposure perturbs fetal brain development through altered placental and fetal immune signaling.

The developing brain is shaped by signals that originate both within and outside the central nervous system (13–15). During pregnancy, the placenta is a major regulator of this communication (16, 17). Rather than functioning only as a passive barrier, the placenta senses the maternal environment and coordinates fetal exposure to inflammatory, endocrine, metabolic, nutrient, and xenobiotic signals (9, 18–24). This placenta–brain axis is especially important during gestation, when neural patterning, vascularization, immune colonization, and tissue remodeling are occurring in parallel (16, 17, 25). Disruption of placental signaling during these windows can therefore alter fetal brain development even when pregnancy outcomes appear relatively normal (26–29). Placental signals can influence fetal brain development through multiple overlapping pathways (17). Changes in placental inflammation, chemokine production, lipid mediator signaling, hormone regulation, nutrient transport, oxidative stress, and vascular function can modify the environment in which the fetal brain develops (16, 25, 30–34). These signals are particularly relevant for microglia, the resident immune cells of the central nervous system (27, 35–37). During fetal development, microglia are still acquiring their mature identity and remain highly sensitive to environmental and immune cues (35–41). Prenatal infection, maternal inflammation, and toxicant exposure can shift microglial phenotype, and these changes may alter how the brain responds to later postnatal challenges (42–46).

One signaling network that may connect placental responses to fetal neuroimmune development is G protein-coupled receptor (GPCR) signaling (47–49). GPCR-linked pathways include chemokine and lipid mediator receptors that regulate immune cell migration, activation, surveillance, and inflammatory tone (50–52). In the developing brain, these pathways are relevant to microglial positioning, maturation, and response to inflammatory cues (53–55). Therefore, disruption of GPCR-linked signaling across the placenta–brain axis represents a plausible mechanism by which prenatal environmental exposures could alter fetal microglial development and postnatal neuroimmune function.

This question is especially important because both placental function and neurodevelopmental vulnerability are strongly modified by biological sex (45, 56–61). Autism spectrum disorder, attention-deficit/hyperactivity disorder, language delay, intellectual disability, and several other neurodevelopmental conditions are diagnosed more frequently in males than females (62–64). Although diagnostic bias and underrecognition in females contribute to these differences, biological sex also influences fetal growth, placental function, immune development, and vulnerability to prenatal stressors (61, 65–67). Male and female fetuses do not respond identically to the same intrauterine environment, and the placenta itself is sexually dimorphic in growth regulation, endocrine signaling, immune tone, stress responses, and xenobiotic handling (9, 61, 68, 69).

Therefore, sex-specific effects of prenatal exposure should not be treated as a secondary observation. Sex-dependent placental, immune, and neurodevelopmental responses to prenatal exposures have been documented across experimental and human studies (9, 42–46, 56). Accordingly, a toxicant may affect both sexes but does so through different developmental trajectories, tissues, or windows of vulnerability. Studies of prenatal benzene exposure should therefore examine male and female offspring separately at defined fetal and postnatal endpoints.

In this study, we tested whether continuous gestational benzene exposure produces sex-dependent changes in placental and fetal brain immune signaling and fetal brain myeloid phenotype. We assessed fetal morphometry, sex-stratified transcriptional profiles, GPCR-associated signaling in the placenta and fetal head/brain, and fetal brain myeloid phenotypes at E12.5 and E17.5. We then evaluated whether prenatal benzene exposure was associated with altered brain responses to postnatal MHV68 infection. Together, these experiments examine neuroimmune outcomes at two fetal collection endpoints and after a defined postnatal viral challenge.

## 2 Materials and methods

### 2.1 Mice and benzene exposure

Male and female C57BL/6 mice (8 to 10 weeks of age) were purchased from The Jackson Laboratory (Bar Harbor, ME) and maintained in a specific pathogen-free animal facility at Wayne State University. Animals were housed under controlled environmental conditions (20 °C to 24°C; 40% to 60% relative humidity) on a 12-h light, 12-h dark cycle (lights on 07:00 to 19:00). Timed matings were performed, and detection of a vaginal plug was designated embryonic day (E) 0.5. Plug-positive females were randomly allocated to one of four filtered air (control) chambers or one of four benzene exposure (5 ppm) chambers (**Figure 1A**). Exposures were carried out continuously (24 h/day) from E0.5 through E12.5 or E17.5 in a whole-body inhalation chamber (CH Technologies, Westwood, NJ), with food and water available ad libitum (9). For fetal endpoints, dams were euthanized at E12.5 or E17.5, and placentas and fetal heads at E12.5 or fetal brains at E17.5 were collected. For postnatal studies, a separate cohort was exposed through E17.5, returned to filtered air, and allowed to deliver naturally. The 5-ppm concentration was selected as a controlled inhalation dose used in developmental benzene studies and in our prior work to identify exposure-associated placental and fetal effects. The design was not intended to reproduce a single typical human exposure scenario. Because the E17.5 cohort received 5 additional days of benzene exposure relative to the E12.5 cohort, comparisons between cohorts reflect developmental age, cumulative exposure duration, and the tissue sampled at each endpoint (whole fetal head at E12.5 versus isolated fetal brain at E17.5). Tissues collected for molecular analyses were snap-frozen in liquid nitrogen and stored at −80°C. Following collection at E12.5 or E17.5, fetal crown-rump length and occipitofrontal diameter were measured as illustrated in **Figure 1A**. Crown-rump length was measured from the crown of the head to the base of the tail, and occipitofrontal diameter was measured across the anterior–posterior axis of the fetal head with a ruler. Fetal sex was determined by quantitative PCR (qPCR) detection of the Y chromosome-associated gene *Ddx3y*. Fetuses producing a positive *Ddx3y* amplification signal were classified as male, whereas fetuses without detectable *Ddx3y* amplification were classified as female (9). Fetal sex was determined before prespecified sex-stratified analyses, and male and female samples were not pooled. Fetal crown-rump length and occipitofrontal diameter were measured on coded specimens by an assessor blinded to the prenatal exposure group. Investigators were not blinded to the prenatal exposure group during sample processing, data acquisition, or downstream analyses for the remaining experiments. All animal procedures were approved by the Wayne State University Institutional Animal Care and Use Committee and conducted in accordance with National Institutes of Health guidelines.

**Figure 1:**
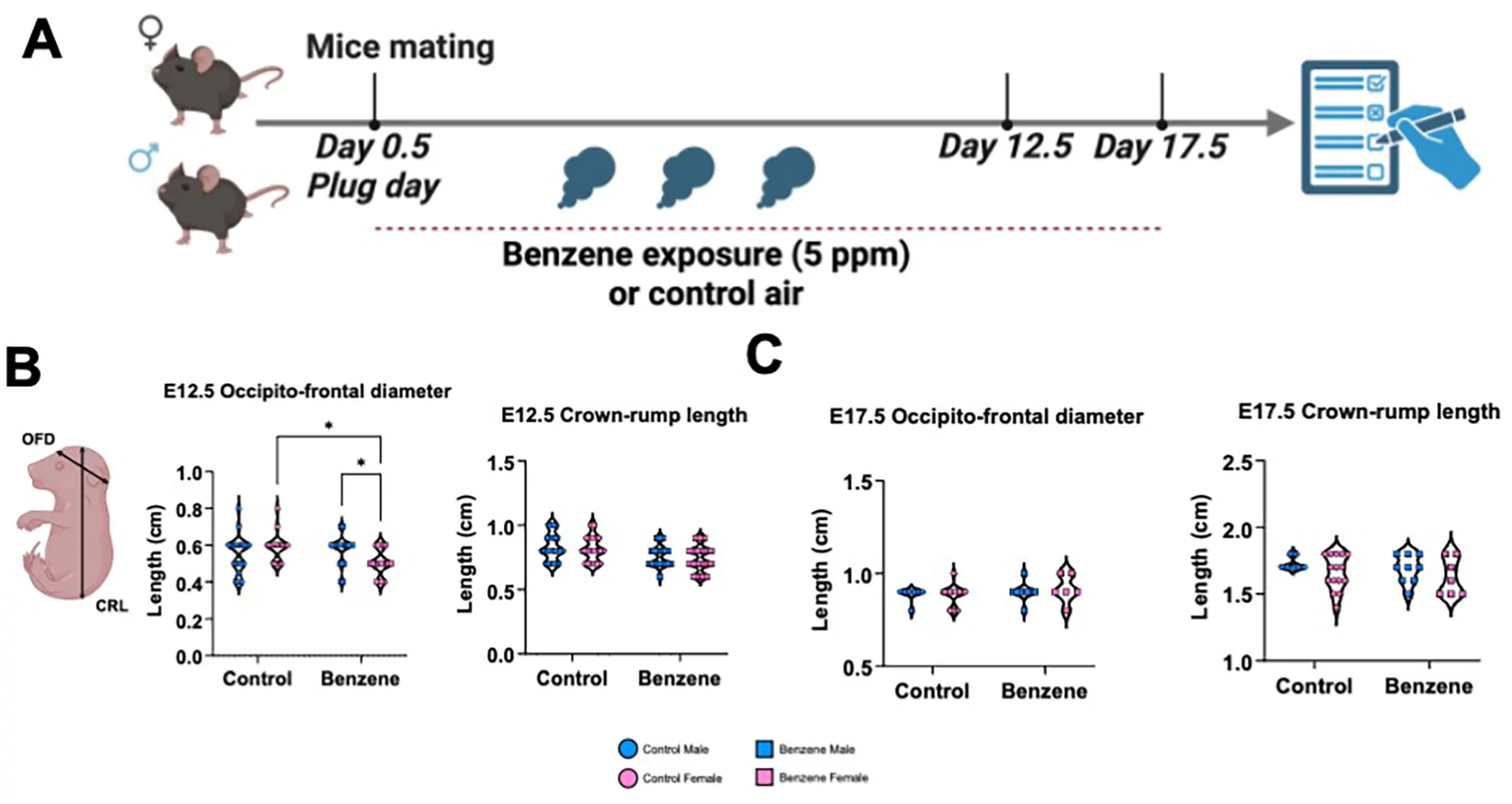
Gestational benzene exposure transiently reduces fetal OFD in a sex-dependent manner. **(A)** Exposure schematic. Plug-positive C57BL/6 dams (E0.5) were exposed to filtered air or 5 ppm benzene continuously until E12.5 or E17.5, followed by collection of fetal tissues and morphometric measurements. **(B)** E12.5 fetal morphometric measurements, including occipitofrontal diameter (OFD) and crown-rump length (CRL), stratified by fetal sex and exposure group. **(C)** E17.5 fetal morphometric measurements, including OFD and CRL, stratified by fetal sex and exposure group. Each data point represents an individual fetus. At E12.5, sample sizes were control males, n = 24; control females, n = 21; benzene-exposed males, n = 20; and benzene-exposed females, n = 19. At E17.5, sample sizes were control males, n = 15; control females, n = 15; benzene-exposed males, n = 9; and benzene-exposed females, n = 8. At each gestational age, fetuses were obtained from four control dams and four benzene-exposed dams, and all available fetuses from each litter were measured; no subset selection was applied. Data in **(B, C)** are displayed as violin plots showing the median (solid line) and the interquartile range (dashed lines). Statistical comparisons were performed using two-way ANOVA followed by Tukey's multiple-comparisons test, where appropriate. P < 0.05 (*).

Sample sizes varied by assay and are reported in the corresponding figure legends. For each endpoint, figure legends specify the number of fetuses or offspring analyzed (n) and the number of contributing dams/litters (N) for each sex- and prenatal-exposure group. Plug-positive dams were randomly allocated to filtered-air or benzene exposure chambers as described above. For fetal morphometric analyses, all available fetuses from each litter were measured. For all other assays, fetal sex was first determined by *Ddx3y* genotyping to permit balanced sex- and exposure-group sampling, and individual fetuses or offspring were then selected at random from within each sex- and prenatal-exposure group, drawn across all contributing litters so that no single dam contributed disproportionately to any group. Sample selection was completed before any outcome measurement was performed.

### 2.2 RNA extraction and quantitative real-time PCR

For mouse tissues, total RNA was isolated using a modified TRIzol-based protocol. Briefly, tissues were homogenized in TRIzol reagent (Ambion, Waltham, MA, USA) with 1.0-mm zirconium beads (Benchmark, Sayreville, NJ, USA) using two 2-min cycles. Homogenates were incubated at room temperature for 5 min, and then chloroform was added at 200 μl per 1 ml TRIzol. Samples were shaken vigorously for 15 s, incubated at room temperature for 3 min, and centrifuged at 12,000 × g for 15 min at 4°C. The aqueous phase was transferred to a fresh tube, and RNA was precipitated by adding 500 μl of 100% isopropanol, incubating at room temperature for 10 min, and centrifuging at 12,000 × g for 10 min at 4°C. The RNA pellet was washed twice with 500 μl of 100% ethanol (12,000 × g for 5 min at 4°C), followed by two washes with 1 ml of 75% ethanol with brief vortexing and centrifugation at 7,500 × g for 5 min at 4°C. Pellets were air-dried for 10 min and resuspended in 50 μl RNase-free water.

RNA concentration and purity were evaluated by A260/A280, and only samples with ratios ≥1.8 were used. For cDNA synthesis, 1 μg total RNA was reverse transcribed using the iScript cDNA Synthesis Kit (Bio-Rad, Hercules, CA, USA). qPCR was performed with iTaq Universal SYBR Green Supermix (Bio-Rad) using gene-specific primers (**Supplementary Table 1**) and cDNA diluted 1:5 in nuclease-free water. Reactions were run on a Bio-Rad CFX96 system (C1000 thermal cycler). Relative expression was calculated using the 2−ΔΔCt method and normalized to housekeeping genes.

### 2.3 MHV68 infection

Offspring from filtered-air- or benzene-exposed pregnancies were inoculated intraperitoneally at postnatal day (PND) 10 with 65 plaque-forming units of murine gammaherpesvirus 68 (MHV68) in a total volume of 50 µl (68). Control offspring received 50 µl of sterile PBS. PND10 was selected as an early postnatal period of active neuroimmune maturation, allowing a defined postnatal immune challenge after prenatal exposure. MHV68 was used as a murine gammaherpesvirus challenge to determine whether prenatal benzene exposure modifies measured brain responses to a later viral challenge. While MHV68 shares similarities to the human gammaherpesvirus, Epstein–Barr virus, it is not intended as a direct model of a specific human neonatal viral infection (70). At 7 days postinfection (PND17), offspring were euthanized and perfused, and brains were collected. ORF65 transcript abundance was quantified by reverse-transcription qPCR and normalized to *Gapdh*.

### 2.4 Statistical analysis

All statistical analyses were performed using GraphPad Prism v8 (GraphPad Software, San Diego, CA). Data are presented as mean ± standard deviation (SD). Normality was evaluated using the Shapiro–Wilk test. When data deviated from normality, it was analyzed using non-parametric approaches, as appropriate. Homogeneity of variance was assessed using Levene’s test. For experiments with two independent factors, group differences and factor interactions were analyzed by two-way ANOVA. When a significant main effect or interaction was detected, *post-hoc* pairwise comparisons were performed using Tukey’s multiple-comparison test. A p value < 0.05 was considered statistically significant.

### 2.5 RNA sequencing and bioinformatics analysis

Total RNA was isolated from fetal tissues as described above. RNA sequencing was performed by the Wayne State University Genomics Core (Detroit, MI, USA). For RNA sequencing, group sizes were n = 6 per sex for E12.5 controls, n = 5 per sex for E12.5 benzene-exposed samples, n = 5 per sex for E17.5 controls, and n = 7 per sex for E17.5 benzene-exposed samples. Libraries were prepared using the Lexogen QuantSeq 3′ mRNA-Seq FWD kit with 12-nt Unique Dual Indexing (UDI) following the manufacturer’s protocol. Library size distribution and quality were evaluated on an Agilent TapeStation (Agilent Technologies, Palo Alto, CA, USA), and library concentration was quantified using a Qubit 2.0 Fluorometer (Thermo Fisher Scientific, Waltham, MA, USA) and qPCR (KAPA Biosystems, Wilmington, MA, USA). Libraries were pooled and sequenced on an Illumina NovaSeq 6000 platform in a single-end format. Imaging and base calling were performed using NovaSeq Control Software. Raw BCL files were converted to FASTQ and demultiplexed using Illumina bcl2fastq v2.17.

Sequencing reads were quality-filtered and trimmed to remove adapters and low-quality bases using BBDUK. Cleaned reads were aligned to the mouse reference genome (GRCm38, Ensembl release) with STAR v2.5.2b. Gene-level counts were generated with HTSeq-count, retaining only uniquely mapped reads assigned to exon features, and a combined count matrix was compiled across all samples.

Differential expression analysis was conducted in R using edgeR. Count data were modeled using a negative binomial framework, and differential expression was tested using generalized linear models with empirical Bayes dispersion estimation and likelihood ratio testing. Differential expression was assessed separately by fetal tissue, developmental stage, and sex for benzene-exposed samples relative to matched controls. Benjamini–Hochberg false discovery rate (FDR) correction was applied. Genes with FDR <0.05 and an absolute log2 fold change >0.6 were considered significantly differentially expressed.

Downstream analyses were performed in R using packages including clusterProfiler, org.Mm.eg.db, AnnotationDbi, enrichplot, ggplot2, dplyr, data.table, and pheatmap. Differentially expressed gene (DEG) lists were annotated with mouse gene identifiers and used for Gene Ontology (GO) Biological Process overrepresentation analysis. Enriched biological processes were identified using clusterProfiler with Benjamini–Hochberg FDR rate correction, and terms with FDR <0.05 were retained for interpretation. Enrichment results were summarized by fetal tissue, developmental stage, and sex, with selected immune- and neurodevelopment-associated biological processes visualized for manuscript figures.

To evaluate receptor- and GPCR-associated transcriptional changes, DEGs were intersected with curated receptor annotations, GO receptor-related annotations, and Reactome/MSigDB GPCR-associated gene sets where applicable. GPCR- and receptor-associated genes were summarized by tissue, developmental stage, and sex, including direction of regulation, overlap across comparisons, and representation among the DEG lists. DEG overlap across comparisons was visualized using overlap summaries, heatmaps, and Venn-style plots. Candidate genes selected for qPCR validation were cross-checked against RNA-seq differential expression tables and incorporated into DEG and receptor-focused visualizations when present.

### 2.6 Flow cytometry

For the PND17 postnatal vehicle- and MHV68-challenge cohorts, offspring were perfused before brain collection using the perfusion buffer described below. Fetal E12.5 head and E17.5 brain samples were collected without perfusion. Offspring were perfused with 5 ml of perfusion buffer (1 ml EDTA in calcium/magnesium-free PBS). Cells from fetal heads, fetal brains, and neonatal offspring brains were dissociated in 0.1% collagenase (C2139, Sigma-Aldrich) for 1 h at 37°C and then passed through a 70-μm filter to obtain single-cell suspension. A Percoll (GE17-0891-01;Cytiva) density gradient was performed. Following Percoll, the cell pellet was resuspended in 3-5 ml of 1× red blood cell lysis buffer (00-4333-57; Thermo Fisher Scientific) and incubated for 10 min on ice and washed with FACS buffer (1× PBS + 1% bovine serum albumin + 0.05% sodium azide). 1×10^6^ cells were resuspended in 100 µl FACS buffer and stained for 30 min for microglia and macrophage markers (**Supplementary Table 2**) after Fc receptor blocking (ab324363; Abcam). After staining, cells were rinsed three times with FACS buffer. Following surface staining, cells were fixed and permeabilized using eBioscience Fixation/Permeabilization working solution prepared by combining one part Fixation/Permeabilization Concentrate with three parts Fixation/Permeabilization Diluent (00-5223-56; Thermo Fisher Scientific). Cells were subsequently stained intracellularly for 30 min at 4 °C (**Supplementary Table 2**). Data were acquired using a CytoFLEX analyzer and CytExpert acquisition software (Beckman Coulter). Data were analyzed, and histograms were generated using FlowJo (Becton, Dickinson and Company). The gating strategy for the analysis of fetal head, fetal brain, and offspring brains is shown in **Supplementary Figure 1**. Raw median fluorescence intensity (MFI) values for each sample were normalized to the median MFI of the corresponding control group and expressed as log_2_ fold change.

## 3 Results

### 3.1 Gestational benzene exposure induces a transient female-specific reduction in fetal head growth

Consistent with our previous observations, benzene exposure did not significantly affect overall pregnancy outcomes (68). To evaluate fetal growth and head development, crown-rump length (CRL) and occipitofrontal diameter (OFD) were measured in male and female fetuses. At E12.5, benzene exposure did not significantly alter CRL in either sex (**Figure 1B**). Similarly, male fetuses showed no differences in OFD compared with sex-matched controls. In contrast, female fetuses exposed to benzene exhibited a significant reduction in OFD despite having normal CRL measurements, indicating a selective effect on head growth rather than overall fetal size (**Figure 1B**).

We next examined whether these morphometric differences persisted later in gestation. By E17.5, neither CRL nor OFD was significantly different between control and benzene-exposed fetuses in either sex (**Figure 1C**). Fetal weight, pregnancy loss, and placental efficiency are reported in a previous study (68). Together, these data indicate that gestational benzene exposure does not cause a broad reduction in fetal linear growth but is associated with a transient, female-specific reduction in OFD at E12.5 that is no longer detected by E17.5.

### 3.2 Prenatal benzene exposure alters fetal neurodevelopmental transcription at two fetal collection endpoints in a sex-dependent manner

Our next objective was to determine if gestational exposure to benzene resulted in transcriptional differences in the fetal brain. This was performed by exposing dams to a similar model as **Figure 1A**. At E12.5 or E17.5, the fetal head or fetal brain, respectively, were collected (**Figure 2A**). Bulk RNA-seq was performed on each of these tissues and stratified by sex. Volcano plot analysis showed that maternal benzene exposure resulted in transcriptional changes in the fetal head (E12.5) and fetal brain (E17.5) (**Figure 2B**). In E12.5 fetal head, prenatal benzene exposure was associated with 405 DEGs in females (141 upregulated, 264 downregulated) and 147 DEGs in males (67 upregulated, 80 downregulated) compared with sex-matched controls (**Figure 2B**). At E17.5 in fetal brain, benzene exposure was associated with 335 DEGs in females (213 upregulated, 122 downregulated) and 1,666 DEGs in males (57 upregulated, 1,609 downregulated) relative to controls (**Figure 2B**). GO biological process enrichment showed that the E12.5 female fetal head was primarily enriched for cell adhesion and muscle- and extracellular matrix-related processes. These included terms such as muscle structure and tissue development, muscle system processes, extracellular structure and extracellular matrix organization, and skeletal muscle development and contraction. In contrast, male fetal head DEGs did not yield any terms that remained significant after FDR correction. Therefore, we reported the top-ranked terms by adjusted p value, which were largely related to GPCR signaling and intercellular communication, including adenylate cyclase-modulating GPCR signaling, gap junction assembly, cardiac conduction-related terms, and CNS myelination/axon ensheathment (**Figure 2C**). At E17.5 in fetal brain, female GO terms were centered around morphogenesis (anatomical structure formation involved in morphogenesis, embryo and organ development, pattern specification, regionalization, and anterior–posterior patterning). In the E17.5 male fetal brain, terms were enriched for cell movement, structural remodeling, circulatory system development, and extracellular structure (**Figure 2D**).

**Figure 2:**
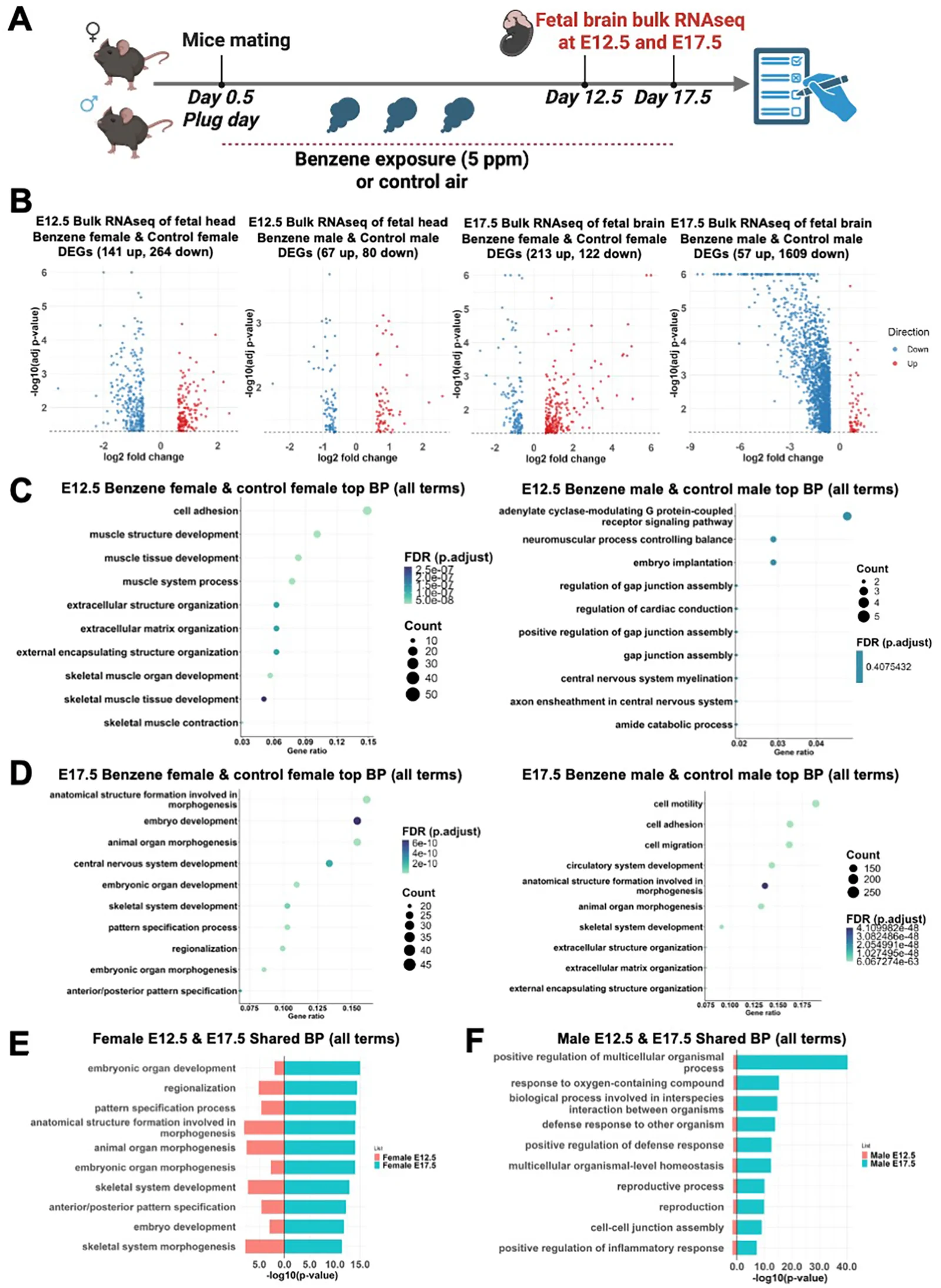
Maternal benzene exposure induces sex-dependent transcriptional remodeling at two fetal collection endpoints in fetal head and fetal brain. **(A)** Tissue collection schematic: E12.5 fetal heads and E17.5 fetal brains were collected following the exposure model in **Figure 1A** and processed for bulk RNA-seq with sex stratification. **(B)** Volcano plots summarizing benzene-associated DEGs in E12.5 fetal head (female and male) and E17.5 fetal brain (female and male). Total DEGs and directionality (up vs. down) are indicated for each comparison. Axis ranges are scaled independently for each panel. **(C)** GO: BP enrichment in E12.5 fetal head (female and male). Dot size reflects gene count and color reflects FDR-adjusted p value. **(D)** GO: BP enrichment DEGs in E17.5 fetal brain (female and male). **(E)** Shared GO: BP terms between E12.5 and E17.5 in females, plotted as −log10(p value) for each time point. **(F)** Shared GO: BP terms between E12.5 and E17.5 in males, plotted as −log10(p value) for each time point.

Lastly, we examined shared GO pathways that were significantly enriched between both time points. Because no E12.5 male terms survived FDR correction, the cross-stage overlap analysis was performed using raw p values and should be considered exploratory. This analysis was used to identify potentially shared biological themes rather than statistically significant cross-stage enrichment. In females, both E12.5 and E17.5 displayed enrichment for fundamental developmental and morphogenesis pathways, including embryonic organ development, regionalization, pattern specification, anatomical structure formation, organ morphogenesis, skeletal development, and anterior–posterior patterning (**Figure 2E**). In contrast, males exhibited shared enrichment for multicellular organismal and host-response processes between E12.5 and E17.5. These included positive regulation of multicellular organismal functions, responses to oxygen-containing compounds, defense responses to other organisms, interspecies interactions, organism-level homeostasis, reproductive processes, cell–cell junction assembly, and positive regulation of inflammatory signaling (**Figure 2F**).

Overall, maternal benzene exposure was associated with sex-dependent transcriptional changes at the E12.5 fetal–head and E17.5 fetal–brain collection endpoints. Females showed developmental and morphogenesis-related enrichment at both endpoints, whereas males showed distinct transcriptomic profiles at E12.5 and E17.5. Because the cohorts differed in developmental age, cumulative exposure duration, and neural tissue sampled, these data do not define a within-tissue developmental trajectory.

### 3.3 Benzene exposure during pregnancy has an impact on brain-associated immunological pathways

We previously showed that maternal benzene exposure impacts key immunological pathways and genes (9). Therefore, we wanted to examine if this held true in the brain from fetuses exposed to benzene. To focus specifically on immune pathways impacted by benzene, we filtered enriched GO terms to only immune-related terms. In females at E12.5, the top enriched immune processes were associated with leukocyte trafficking programs, including leukocyte migration and chemotaxis and leukocyte regulation. Terms associated with erythrocyte physiology and behavioral responses were also enriched (**Figure 3A**). At E17.5, female immune enrichment shifted toward broader immune and hematopoietic development pathways. These terms included regulation of molecular mediator production during immune responses, immune system and lymphoid organ development, thymus and bone marrow development, and neuroinflammatory and glial activation programs (**Figure 3A**). For males at E12.5, immune GO enrichment did not yield any terms that remained significant after FDR correction. Therefore, we report the top-ranked immune terms by adjusted p value. These terms were largely centered on host defense and early inflammatory signaling, including defense response to other organisms, acute inflammatory and acute-phase responses, and regulation of pattern-recognition pathways such as MDA-5 and TLR2 signaling (**Figure 3A**). At E17.5, males showed enrichment for core immune activation programs, including defense response, inflammatory response, regulation and positive regulation of immune system processes, immune effector processes, and leukocyte migration (**Figure 3A**).

**Figure 3:**
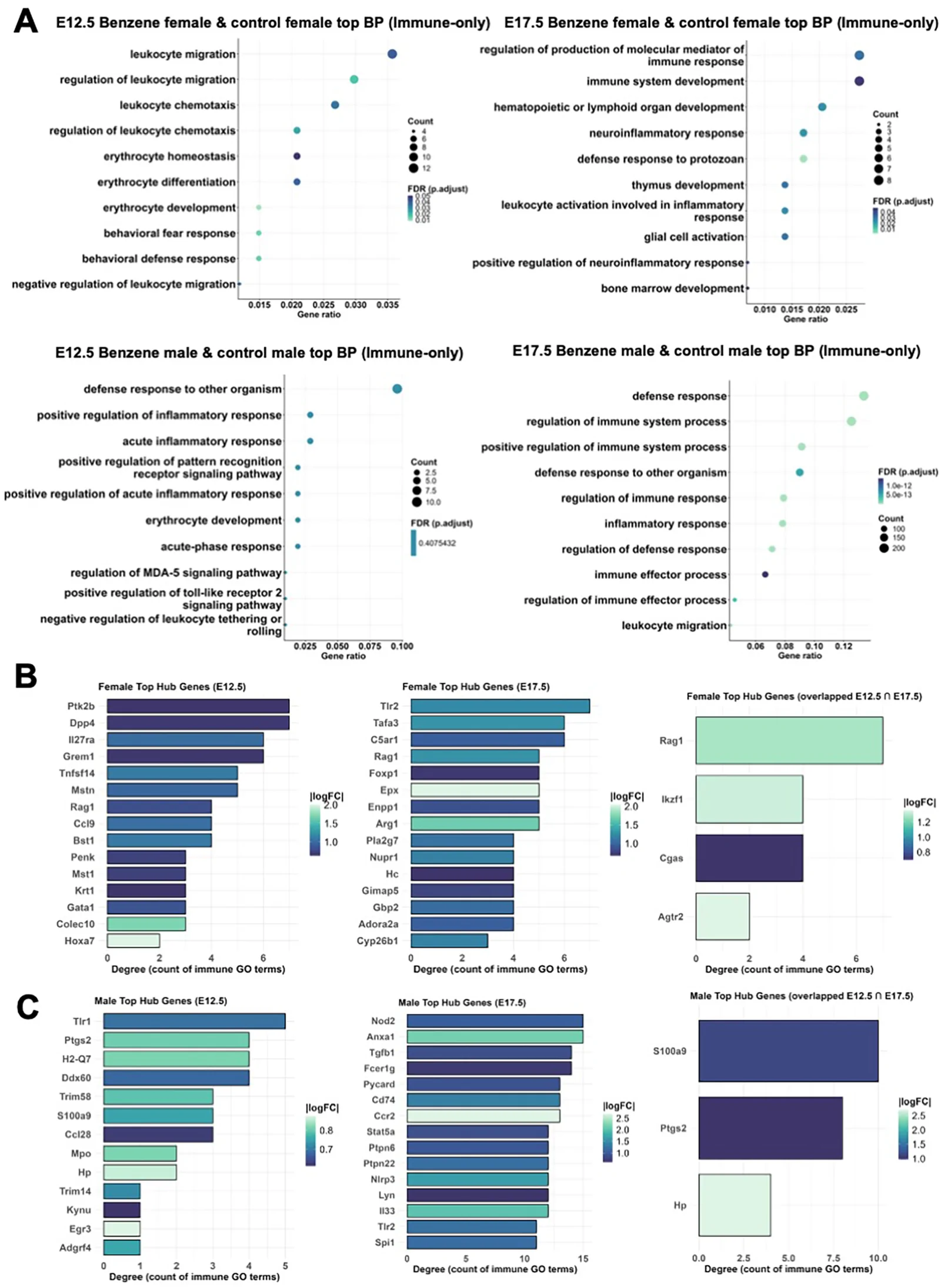
Immune-focused pathway and hub-gene analyses identify sex-dependent immune signatures at two fetal collection endpoints. **(A)** Immune-only GO: BP enrichment after subsetting enriched GO terms to immune-related processes for E12.5 fetal head (female and male) and E17.5 fetal brain (female and male). Dot size reflects gene count and color reflects FDR-adjusted p value. **(B)** Female immune-network hub genes at E12.5 and E17.5 ranked by degree (number of immune GO terms mapped to each gene), with the subset of overlapping hubs between E12.5 and E17.5 shown at right. Bar length indicates degree and color indicates |logFC|. **(C)** Male immune-network hub genes at E12.5 and E17.5 ranked by degree, with overlapping hubs between E12.5 and E17.5 shown at right. Bar length indicates degree and color indicates |logFC|.

Next, we asked which genes were most central across the immune GO networks by ranking hubs based on degree (the number of immune GO terms each gene mapped to) and identified hubs shared between E12.5 and E17.5 (**Figures 3B, C**). In females in the E12.5 fetal head, the highest-degree hubs were found to be *Ptk2b*, *Dpp4*, *Il27ra*, *Grem1*, and *Tnfsf14* (**Figure 3B**). In the E17.5 fetal brain, the female hub structure shifted toward lymphoid and antigen-related pathways (**Figure 3B**). Top hubs included *Tlr2*, *Tafa3*, *C5ar1*, *Rag1*, and *Foxp1* (**Figure 3B**). This also included a set of immune activation genes, such as *Gbp2*, *Gimap5*, and *Cyp26b1* (**Figure 3B**). When we then looked for hubs shared at E12.5 and E17.5 in females, overlap here was mainly associated with *Rag1*, but *Ikzf1*, *Cgas*, and *Agtr2* were also retained as shared nodes to a lesser degree (**Figure 3B**).

Hubs in the male E12.5 fetal head were largely associated with innate immune sensing and inflammatory signaling genes, including *Tlr1*, *Ptgs2*, H2-Q7, *Ddx60*, and *Trim58* (**Figure 3C**). At E17.5, hubs in the male fetal brain were shifted toward inflammation and myeloid activation (**Figure 3C**). High-degree nodes here included *Nod2*, *Anxa1*, *Tgfb1*, *Fcer1g*, *Pycard*, and *Cd74*. Another set of hubs were associated with chemokine signaling, which included *Ccr2*, *Stat5a*, *Ptpn6*, *Nlrp3*, and *Spi1* (**Figure 3C**). Overlap of male hub genes across E12.5 and E17.5 was limited. Shared hubs were *S100a9*, *Ptgs2*, and *Hp*, which suggests an inflammatory phenotype between the observed endpoints (**Figure 3C**).

Taken together, these analyses show that prenatal benzene exposure was associated with sex-dependent immune transcriptional signatures that differed between the E12.5 fetal–head and E17.5 fetal–brain collection cohorts. Females are shifted from leukocyte trafficking signals at E12.5 toward immune and hematopoietic developmental and neuroinflammatory programs at E17.5, whereas males display an overlapped signature around host-defense and inflammatory activation.

### 3.4 Sex-biased communication between the placenta and the developing brain

Given the immune and inflammatory signatures identified in the fetal head and brain, we next asked whether benzene exposure altered receptor-linked signaling pathways that could connect placental responses with fetal neurodevelopmental changes. We first summarized benzene-responsive genes by receptor signaling class in the placenta and fetal head/brain at E12.5 and E17.5. Across both tissues and developmental time points, GPCR/chemokine signaling was repeatedly represented among the receptor-associated gene programs altered by benzene exposure (**Figure 4A**). However, the direction of this response was not uniform. Instead, GPCR/chemokine signaling showed patterns that differed by tissue, fetal sex, and collection endpoint.

**Figure 4:**
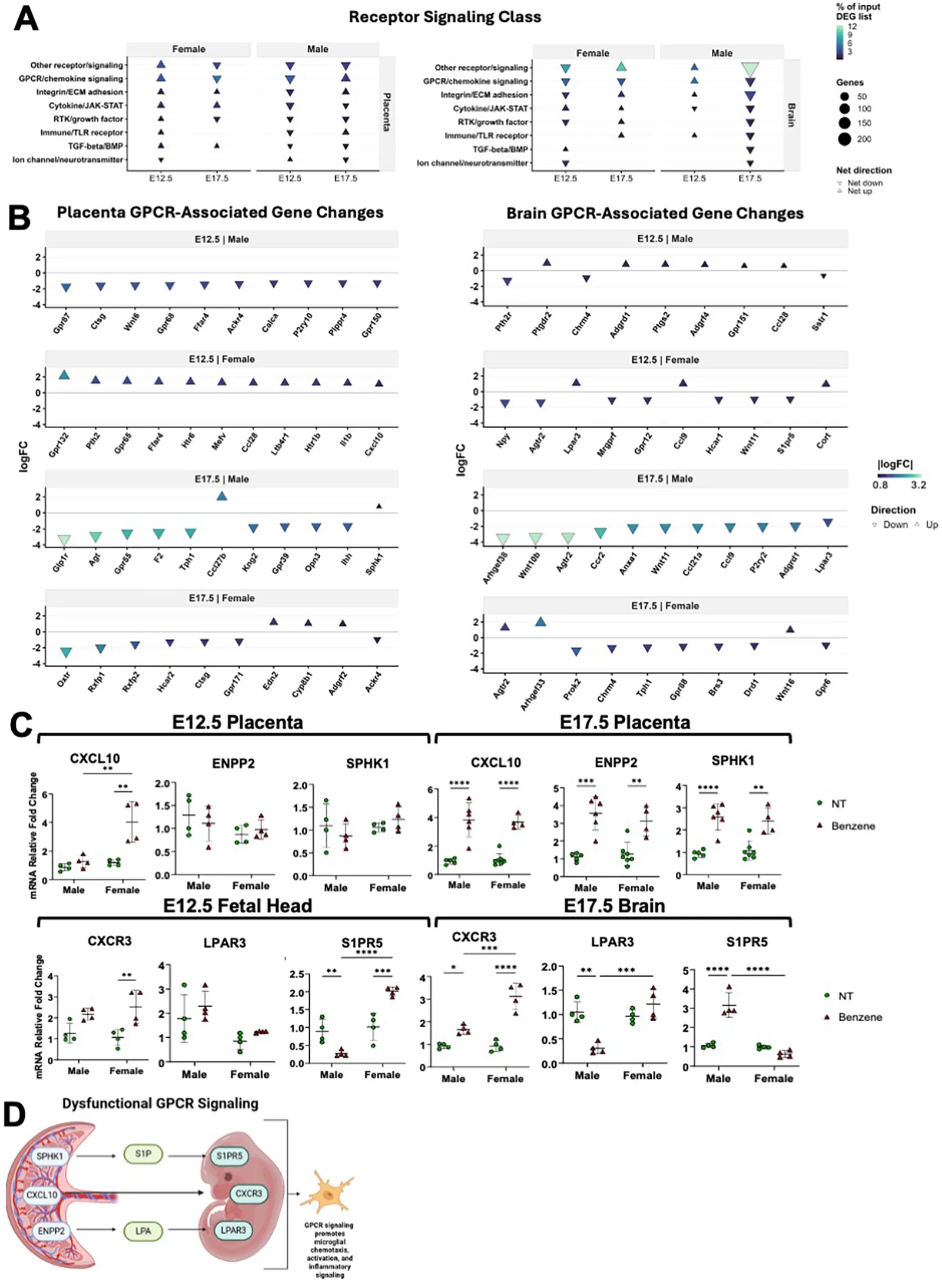
Gestational benzene exposure disrupts GPCR-linked signaling across the placenta and fetal head/brain. **(A)** Receptor signaling class analysis of benzene-responsive genes in placenta and fetal head/brain at E12.5 and E17.5, stratified by fetal sex. GPCR/chemokine signaling was identified as a recurrent receptor-associated pathway altered by gestational benzene exposure. **(B)** Directional point plot of GPCR-associated gene changes in placenta and fetal head/brain following benzene exposure. Placental GPCR-associated gene changes are shown at E12.5 and E17.5 by fetal sex. Fetal head/brain GPCR-associated gene changes are shown at E12.5 fetal head and E17.5 fetal brain by fetal sex. Color scale represents log2 fold change in benzene-exposed samples relative to sex-matched controls. **(C)** qPCR validation of GPCR-linked placental mediators and fetal head/brain receptor targets. Placental expression of *Cxcl10*, *Enpp2*, and *Sphk1* was measured at E12.5 and E17.5. Expression of the corresponding fetal head/brain receptor targets *Cxcr3*, *Lpar3*, and *S1pr5* was measured in E12.5 fetal head and E17.5 fetal brain, stratified by fetal sex and exposure group. **(D)** Proposed model linking gestational benzene exposure to altered placenta-to-brain GPCR-associated signaling. Benzene exposure disrupts placental chemokine and lipid mediator pathways, including CXCL10-CXCR3, ENPP2-LPA-LPAR3, and SPHK1-S1P-S1PR5 signaling, which may contribute to altered fetal brain myeloid/microglial programming. For qPCR panels, each data point represents an individual placenta or fetal tissue sample. For E12.5 placental analyses, n = 4 samples per sex and exposure group, obtained from N = 2 dams per exposure condition. For E17.5 placental analyses, sample sizes were control males, n = 5; benzene-exposed males, n = 6; control females, n = 7; and benzene-exposed females, n = 4, obtained from N = 3 dams per exposure condition. Fetal sex was determined by *Ddx3y* genotyping prior to sample selection, and placental and fetal samples were then selected at random from within each sex- and exposure group, drawn across the contributing dams (Section 2.1). Data are presented as mean ± SD. Statistical comparisons were performed using two-way ANOVA with Tukey’s *post-hoc* test where appropriate. P < 0.05 (*), P < 0.01 (**), P < 0.001 (***), and P < 0.0001 (****).

We next examined the individual GPCR-associated genes contributing to this signal in the placenta and fetal head/brain. In the placenta, the E12.5 response was strongly sex divergent. Male placentas showed a broadly downregulated GPCR-associated profile, including decreased expression of *Gpr87, Ctsg, Wnt6, Gpr68, Ffar4, Ackr4, Calca, P2ry10, Plppr4*, and *Gpr150* (**Figure 4B**). In contrast, female placentas at E12.5 showed the opposite pattern, with GPCR-associated genes predominantly increased. This included *Gpr132, Pth2, Gpr65, Ffar4, Htr6, Mefv, Ccl28, Ltb4r1, Htr1b, Il1b*, and *Cxcl10* (**Figure 4B**). Thus, at early gestation, benzene exposure was associated with suppression of GPCR-associated genes in male placentas, whereas female placentas showed activation of chemokine-, inflammatory-, and receptor-linked genes.

By E17.5, placental GPCR-associated disruption remained sex dependent, but the pattern shifted. Male placentas showed strong downregulation of several GPCR-related genes, including *Glp1r, Agt, Gpr55, F2*, and *Tph1*, whereas *Ccl27b* and *Sphk1* were increased (**Figure 4B**). Female placentas at E17.5 also showed a mixed response, with decreased expression of *Oxtr, Rxfp1, Rxfp2, Hcar2, Ctsg, Gpr171*, and *Ackr4*, alongside increased expression of *Edn2, Cyp8b1*, and *Adgrf2* (**Figure 4B**). These data indicate that placental GPCR-associated transcription differed between the E12.5 and E17.5 collection cohorts. The affected genes and direction of change also varied by fetal sex.

A similar pattern, differing by fetal sex and collection endpoint, was observed in the fetal head and brain. At E12.5, male fetal heads showed a mixed GPCR-associated response, with decreased *Pth2r, Chrm4*, and *Sstr1* but increased *Ptger2, Adgrd1, Ptgs2, Adgrf4, Gpr151*, and *Ccl28* (**Figure 4B**). In contrast, female fetal heads at E12.5 showed downregulation of several GPCR-associated genes, including *Npy, Agtr2, Mrgprf, Gpr12, Hcar1, Wnt11*, and *S1pr5*, whereas *Lpar3, Ccl9*, and *Cort* were increased (**Figure 4B**). By E17.5, the male fetal brain exhibited a broad downregulated GPCR-associated profile, including *Arhgef38, Wnt10b, Agtr2, Ccr2, Anxa1, Wnt11, Ccl21a, Ccl9, P2ry2, Adgrd1*, and *Lpar3* (**Figure 4B**). Female fetal brains at E17.5 showed a different pattern, with increased *Agtr2, Arhgef33*, and *Wnt16* but decreased *Prok2, Chrm4, Tph1, Gpr88, Brs3, Drd1*, and *Gpr6* (**Figure 4B**). Together, these findings show that benzene exposure was associated with distinct GPCR-associated transcriptional profiles in placenta and fetal neural tissues at the two collection endpoints. The affected genes and directionality differed by fetal sex.

Based on these pathway-level and gene-level findings, we selected a focused set of GPCR-linked mediators for validation by qPCR. We prioritized GPCR-associated placental ligands and lipid mediator enzymes, including *Cxcl10*, *Enpp2*, and *Sphk1*, along with corresponding fetal head/brain receptor targets *Cxcr3*, *Lpar3*, and *S1pr5*. At E12.5, benzene exposure significantly increased placental *Cxcl10* expression in females, whereas *Enpp2* and *Sphk1* were not significantly altered in either sex (**Figure 4C**). By E17.5, this placental response was broader, with *Cxcl10, Enpp2*, and *Sphk1* all significantly increased in benzene-exposed placentas from both male and female fetuses (**Figure 4C**). These findings show distinct placental GPCR-linked qPCR patterns at the E12.5 and E17.5 collection endpoints. Because the cohorts differed in developmental age, cumulative exposure duration, and tissue sampled, these data do not establish a progressive increase across gestation.

We then examined the expression of the corresponding receptor targets in the fetal head at E12.5 and fetal brain at E17.5. At E12.5, *Cxcr3* was significantly increased only in benzene-exposed female fetal heads, whereas *Lpar3* was not significantly altered in either sex (**Figure 4C**). In contrast, *S1pr5* showed a divergent sex-specific response, with decreased expression in benzene-exposed male fetal heads and increased expression in benzene-exposed female fetal heads (**Figure 4C**). At E17.5, *Cxcr3* expression was significantly increased in both male and female fetal brains following benzene exposure, with the strongest induction observed in females. *Lpar3* was significantly reduced in benzene-exposed male fetal brains, whereas females showed no significant change. *S1pr5* showed the opposite pattern, with a significant increase in benzene-exposed male fetal brains and no significant change in females (**Figure 4C**).

Together, these results indicate that gestational benzene exposure disrupts GPCR-linked signaling across the placenta and fetal head/brain. The qPCR validation supports a possible model in which placental chemokine and lipid mediator pathways, including CXCL10-CXCR3, ENPP2-lysophosphatidic acid (LPA)-LPAR3, and SPHK1- sphingosine-1-phosphate (S1P)-S1PR5 signaling, are altered in parallel with fetal brain receptor expression (**Figure 4D**) (50). These data nominate GPCR signaling as a potential placenta-to-brain communication axis through which benzene exposure may influence microglial recruitment, activation, chemotaxis, and inflammatory programming during fetal development.

### 3.5 Gestational benzene exposure alters the phenotype of fetal brain myeloid/microglia-enriched cells

Because GPCR-linked chemokine and lipid mediator pathways regulate myeloid cell trafficking, activation, and tissue surveillance (50, 52, 71), we next asked whether the GPCR-associated transcriptional changes identified in the placenta and fetal head/brain were accompanied by changes in fetal brain myeloid cells. We analyzed E12.5 fetal head and E17.5 fetal brain CD45 ^low/int^ CD11b^+^ cells and quantified IBA1 mean fluorescence intensity (MFI) within this population as a marker of the myeloid/microglia-enriched phenotype (72). We also examined EGR2 and MHC II expression within the CD45^low/int^ CD11b^+^ IBA1^+^ population, where EGR2 is associated with regulatory and maturation-linked myeloid programs and MHC II reflects antigen presentation and inflammatory activation (73, 74).

At E12.5, benzene exposure did not significantly alter the IBA1 MFI in CD45 ^low/int^ CD11b^+^ cells in either male or female fetal heads, suggesting that early gestational benzene exposure did not broadly change the IBA1 expression within the total brain myeloid compartment at this time point (**Figure 5A**). The CD45 MFI in singlets and the CD11b MFI in CD45 ^low/int^ cells were also not consistently altered by benzene exposure at E12.5 (**Supplementary Figure 2**). The EGR2 MFI within CD45 ^low/int^ CD11b^+^ IBA1^+^ cells was also not significantly changed by benzene exposure at E12.5, although males showed a trend toward reduced EGR2 expression following exposure (**Figure 5B**). MHC II expression exhibited modest sex-dependent differences at E12.5. Benzene-exposed male fetal heads showed a significantly lower MHC II MFI within CD45 ^low/int^ CD11b^+^ IBA1^+^ cells, compared with benzene-exposed female fetal heads (**Figure 5C**). Overall, these findings indicate that early benzene exposure induces only minor changes in fetal head myeloid/microglial phenotype at E12.5, with limited evidence for alterations in antigen-presentation-associated markers.

**Figure 5:**
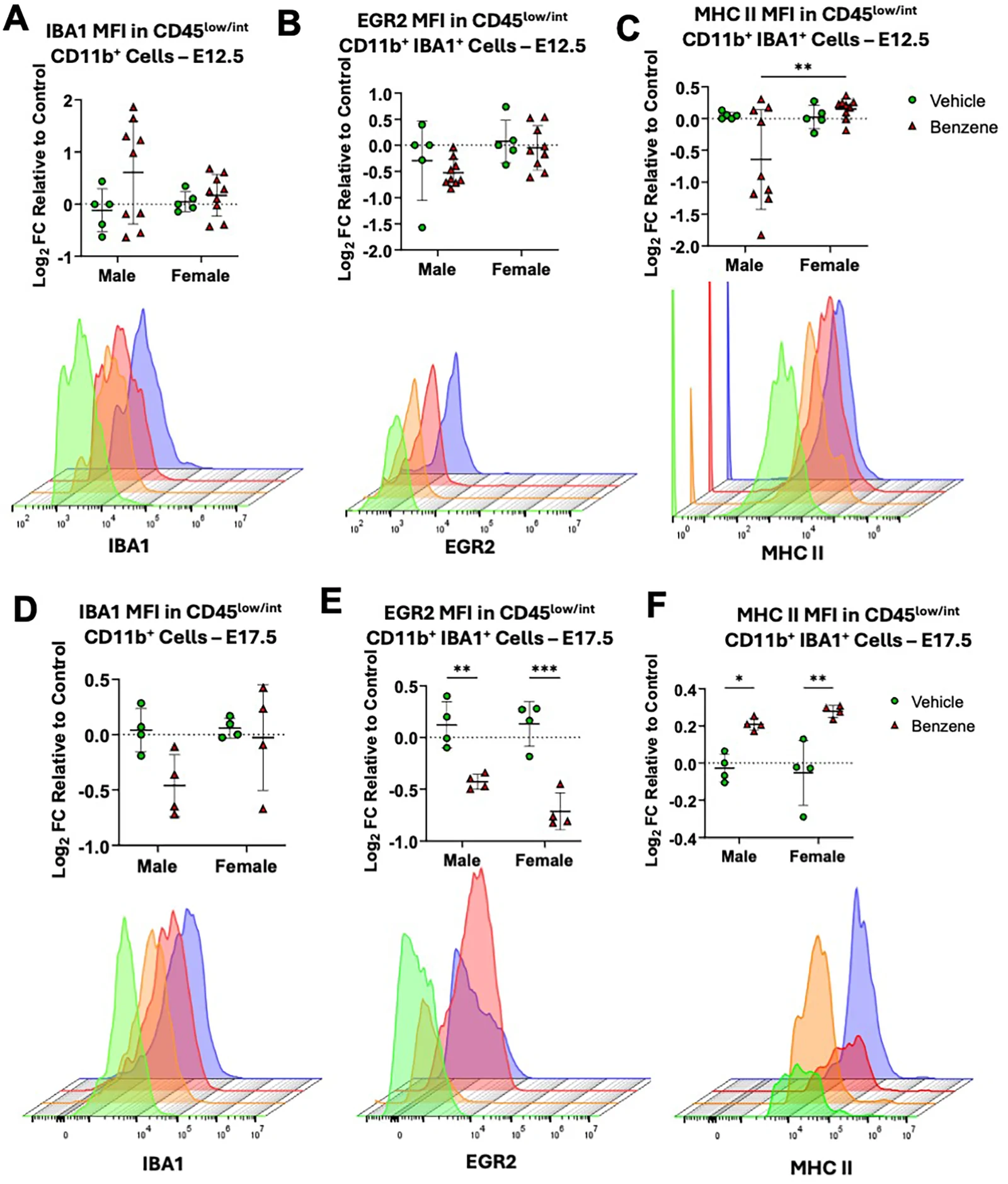
Prenatal benzene exposure alters myeloid/microglia-enriched phenotypes at two fetal collection endpoints. **(A)** IBA1 mean fluorescence intensity (MFI) in CD45^low/int^ CD11b^+^ fetal head myeloid cells at E12.5, stratified by fetal sex and exposure group. **(B)** EGR2 MFI in CD45^low/int^ CD11b^+^ IBA1^+^ fetal head myeloid/microglia-enriched cells at E12.5, stratified by fetal sex and exposure group. **(C)** MHC II MFI in CD45^low/int^ CD11b^+^ IBA1^+^ fetal head myeloid/microglia-enriched cells at E12.5, stratified by fetal sex and exposure group. **(D)** IBA1 MFI in CD45^low/int^ CD11b^+^ fetal brain myeloid cells at E17.5, stratified by fetal sex and exposure group. **(E)** EGR2 MFI in CD45^low/int^ CD11b^+^ IBA1^+^ fetal brain myeloid/microglia-enriched cells at E17.5, stratified by fetal sex and exposure group. **(F)** MHC II MFI in CD45^low/int^ CD11b^+^ IBA1^+^ fetal brain myeloid/microglia-enriched cells at E17.5, stratified by fetal sex and exposure group. Representative histogram overlays are shown for IBA1, EGR2, and MHC II expression where indicated. Each data point represents an individual fetus. At E12.5, sample sizes were control males, n = 5; control females, n = 5; benzene-exposed males, n = 9; and benzene-exposed females, n = 9. Control samples were obtained from N = 3 litters, and benzene-exposed samples were obtained from N = 4 litters. At E17.5, n = 4 fetuses were analyzed per sex and exposure group, obtained from N = 3 dams/litters per exposure condition. Fetal sex was determined by *Ddx3y* genotyping prior to sample selection, and fetuses were then selected at random from within each sex- and exposure group, drawn across the contributing litters so that no single dam contributed disproportionately to any group. Data are presented as mean ± SD. Statistical comparisons were performed using two-way ANOVA followed by Tukey’s multiple-comparisons test, where appropriate. P < 0.05 (*), P < 0.01 (**), and P < 0.001 (***).

We next examined whether further changes were observed by E17.5. Similar to E12.5, the IBA1 MFI in CD45 ^low/int^ CD11b^+^ cells was not significantly altered by benzene exposure in either sex at E17.5 (**Figure 5D**). The CD45 MFI in singlets and the CD11b MFI in CD45 ^low/int^ cells were also not consistently altered by benzene exposure at E17.5 (**Supplementary Figure 3**). However, the EGR2 MFI was significantly reduced in CD45 ^low/int^ CD11b^+^ IBA1^+^ cells from both male and female benzene-exposed fetal brains (**Figure 5E**). This reduction suggests that later benzene exposure through E17.5 is associated with a loss of EGR2-associated regulatory or maturation-linked myeloid features. In parallel, the MHC II MFI was significantly increased in CD45 ^low/int^ CD11b^+^ IBA1^+^ cells from both male and female benzene-exposed fetal brains (**Figure 5F**). Representative histogram overlays supported these shifts in marker intensity following benzene exposure at E17.5.

Together, these results show that the effects of prenatal benzene exposure on myeloid/microglia-enriched phenotypes differed between the E12.5 fetal–head and E17.5 fetal–brain collection cohorts. At E12.5, the response is most evident as a sex-divergent change in MHC II expression, whereas by E17.5, benzene exposure produces a more consistent reduction in EGR2 and induction of MHC II across both sexes. These findings may connect the GPCR-associated transcriptional changes identified in **Figure 4** with altered fetal brain myeloid cell state, supporting a possible model in which prenatal benzene exposure shifts microglia-enriched cells away from a regulatory/maturation-associated phenotype and toward an antigen-presentation-associated inflammatory phenotype during fetal brain development.

### 3.6 Prenatal benzene exposure alters postnatal brain immune responses in a sex-specific manner

We next asked whether the fetal brain myeloid changes observed after gestational benzene exposure were associated with altered offspring responses to viral infection. To test this, dams were exposed to filtered air or 5 ppm benzene during pregnancy, and offspring were infected with MHV68 at PND10. Brain tissue was collected at PND17 to quantify ORF65 transcript abundance as a measure of viral gene expression, inflammatory gene expression, and brain myeloid/microglial phenotype (**Figure 6A**).

**Figure 6:**
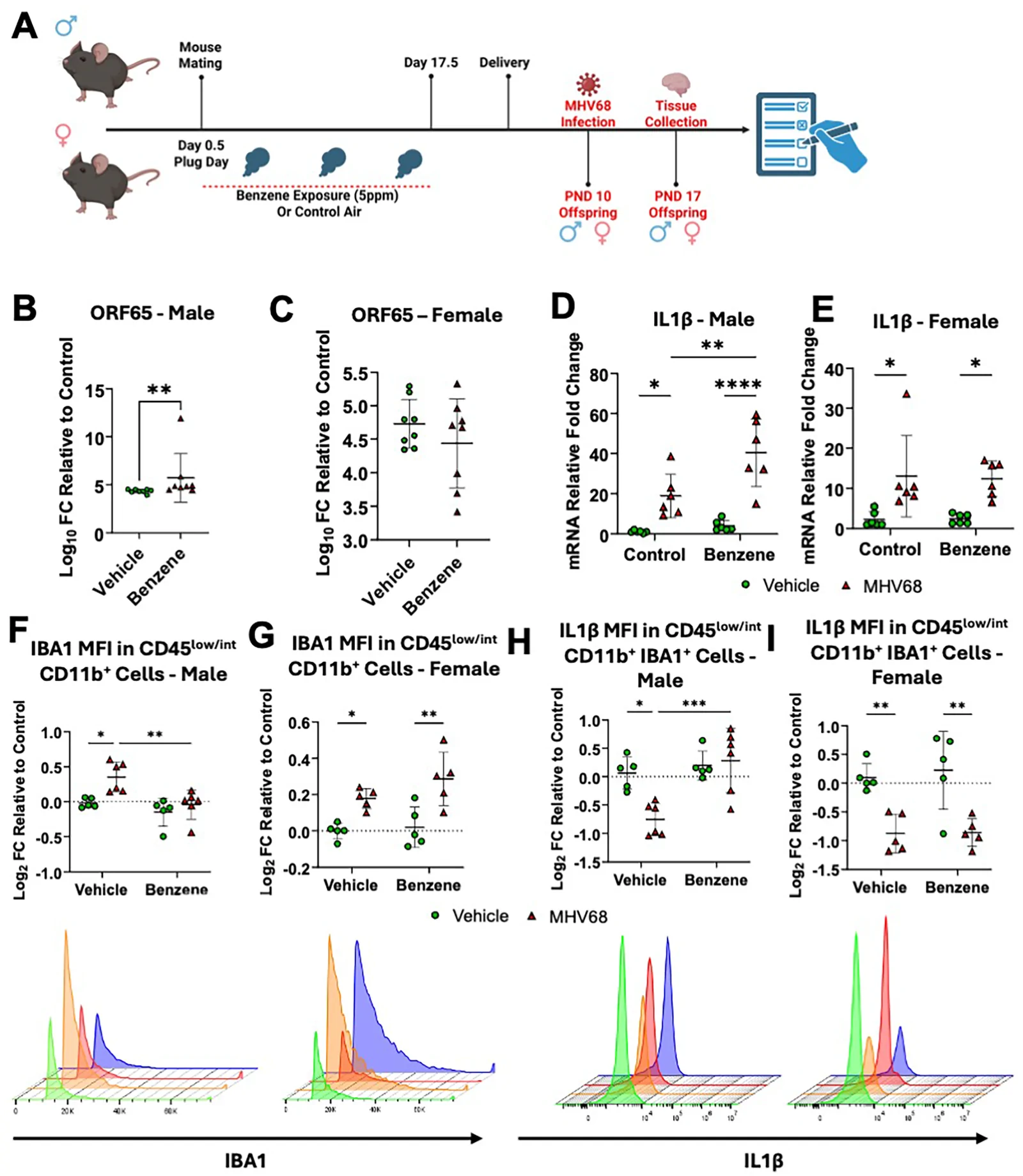
Gestational benzene exposure alters postnatal brain immune responses to MHV68 infection. **(A)** Experimental schematic. Dams were exposed to filtered air or 5 ppm benzene during pregnancy. Offspring were challenged with MHV68 at PND10, and brain tissue was collected at PND17 to assess viral gene expression, inflammatory gene expression, and brain myeloid/microglial phenotype. **(B)** MHV68 ORF65 expression in male offspring brain at PND17, comparing MHV68-infected offspring from control and benzene-exposed pregnancies. **(C)** MHV68 ORF65 expression in female offspring brain at PND17, comparing MHV68-infected offspring from control and benzene-exposed pregnancies. **(D)** *Il1b* expression in male offspring brain at PND17 following prenatal benzene exposure and postnatal MHV68 infection. **(E)** *Il1b* expression in female offspring brain at PND17 following prenatal benzene exposure and postnatal MHV68 infection. **(F)** IBA1 MFI in CD45^low/int^ CD11b^+^ brain myeloid cells from male offspring at PND17 following prenatal benzene exposure and postnatal MHV68 infection. **(G)** IBA1 MFI in CD45^low/int^ CD11b^+^ brain myeloid cells from female offspring at PND17 following prenatal benzene exposure and postnatal MHV68 infection. **(H)** IL-1β MFI in CD45^low/int^ CD11b^+^ IBA1^+^ brain myeloid/microglia-enriched cells from male offspring at PND17 following prenatal benzene exposure and postnatal MHV68 infection. **(I)** IL-1β MFI in CD45^low/int^ CD11b^+^ IBA1^+^ brain myeloid/microglia-enriched cells from female offspring at PND17 following prenatal benzene exposure and postnatal MHV68 infection. Representative histogram overlays are shown for IBA1 and IL-1β expression where indicated. Each data point represents an individual offspring. On the x-axis, Vehicle and Benzene denote the prenatal exposure group (filtered air and 5 ppm benzene); in **(D–I)**, marker symbols denote the postnatal challenge (vehicle, sterile PBS; MHV68). For MHV68 ORF65 analyses **(B, C)**, n = 8 infected offspring were analyzed per prenatal exposure group for each sex. For brain *IL1b* expression **(D, E)**, n = 6 offspring were analyzed per prenatal exposure and postnatal challenge group for each sex. For male flow-cytometric analyses **(F, H)**, sample sizes were control-vehicle, n = 5; control-infected, n = 6; benzene-vehicle, n = 5; and benzene-infected, n = 6. For female flow-cytometric analyses **(G, I)**, n = 5 offspring were analyzed per prenatal exposure and postnatal challenge group. Within each prenatal exposure group, offspring were obtained from three independent dams/litters and were randomly assigned to the postnatal vehicle or MHV68 challenge groups within each sex, drawn across the contributing litters. Data are presented as mean ± SD. Comparisons between the two groups in **(B, C)** were performed using unpaired two-tailed t-tests. Comparisons in **(D–I)** were performed using two-way ANOVA followed by Tukey’s multiple-comparisons test. P <0.05 (*), P <0.01 (**), P <0.001 (***), and P <0.0001 (****).

We quantified MHV68 ORF65 transcript abundance in PND17 brain tissue. In male offspring, prenatal benzene exposure was associated with higher ORF65 transcript abundance after MHV68 infection than in infected offspring from control pregnancies (**Figure 6B**). In females, ORF65 transcript abundance was not significantly different between control and benzene-exposed offspring at PND17 (**Figure 6C**). Thus, the benzene-associated difference observed in this experiment was limited to ORF65 transcript abundance in male brains at the endpoint examined; it does not establish differences in viral replication, clearance, tissue tropism, or the overall post-viral response in either sex.

We next examined whether this increased viral signal was accompanied by altered inflammatory responses in the brain. In males, MHV68 infection increased the *Il1b* expression in offspring from control pregnancies, and this response was markedly amplified in offspring prenatally exposed to benzene (**Figure 6D**). Benzene-exposed male offspring infected with MHV68 showed significantly higher *Il1b* expression than infected male offspring from control pregnancies, indicating that prenatal benzene exposure heightened the postnatal neuroinflammatory response to viral infection. In females, MHV68 infection also increased the *Il1b* expression in both control and benzene-exposed offspring (**Figure 6E**). However, unlike males, prenatal benzene exposure did not further increase the *Il1b* response to infection in females. Together, these data indicate that prenatal benzene exposure produces a male-biased increase in viral gene expression and inflammatory transcriptional response after postnatal MHV68 challenge.

Because fetal benzene exposure altered the brain myeloid/microglial phenotype at E17.5, we next tested whether postnatal infection revealed benzene-associated changes in this compartment. In male offspring from control pregnancies, MHV68 infection increased the IBA1 MFI within CD45 ^low/int^ CD11b^+^ brain myeloid cells (**Figure 6F**). However, this infection-induced increase in IBA1 was blunted in males prenatally exposed to benzene, with MHV68-infected benzene-exposed males showing a significantly lower IBA1 MFI than MHV68-infected control males (**Figure 6F**). This suggests that prenatal benzene exposure alters the postnatal brain myeloid response to viral infection in males, despite the presence of increased viral gene expression and *Il1b* expression. In females, MHV68 infection increased the IBA1 MFI in both control and benzene-exposed offspring (**Figure 6G**), indicating that infection-induced IBA1 upregulation remained intact in females regardless of prenatal benzene exposure. In parallel, MHV68 infection increased the CD45 MFI in singlets and the CD11b MFI in CD45^low/int^ cells in both male and female offspring, supporting a broader brain myeloid response to viral challenge (**Supplementary Figure 4**).

We then quantified the IL-1β expression within the CD45 ^low/int^ CD11b^+^ IBA1^+^ microglia-enriched population. In male offspring from control pregnancies, the MHV68 infection reduced the IL-1β MFI within CD45 ^low/int^ CD11b^+^ IBA1^+^ cells (**Figure 6H**). This reduction was not observed to the same extent in benzene-exposed males, and MHV68-infected benzene-exposed males showed a significantly higher IL-1β MFI than MHV68-infected control males (**Figure 6H**). In females, MHV68 infection reduced the IL-1β MFI within CD45 ^low/int^ CD11b^+^ IBA1^+^ cells in both control and benzene-exposed offspring (**Figure 6I**), with no clear benzene-dependent difference in the infection response. These findings suggest that prenatal benzene exposure modifies the relationship between viral infection, inflammatory gene expression, and microglial IL-1β response in a sex-dependent manner.

Overall, these results extend the fetal findings into the postnatal period and show that gestational benzene exposure was associated with selected postnatal brain immune responses after MHV68 challenge. Prenatal benzene exposure was associated with higher ORF65 transcript abundance at PND17 and higher brain *Il1b* expression in male offspring, while also altering infection-induced IBA1 and IL-1β responses in brain myeloid/microglia-enriched cells. Together with the fetal transcriptional, GPCR signaling, and microglial phenotype data, these findings support a model in which prenatal benzene exposure programs the developing brain immune environment and changes how offspring respond to postnatal viral challenge.

## 4 Discussion

This study identifies gestational benzene exposure as a modifier of fetal brain immune programming and measured postnatal neuroimmune responses. During continuous fetal exposure, benzene was associated with changes in fetal head and brain transcriptional profiles, GPCR-linked signaling, and EGR2/MHC II expression in brain myeloid/microglia-enriched cells. In the postnatal MHV68 experiment, benzene-associated differences at PND17 were observed in selected male outcomes, including higher ORF65 and *Il1b* transcript abundance and altered IBA1/IL-1β responses. These data support the interpretation that prenatal benzene exposure alters measured neuroimmune endpoints across development. They do not establish the presence or absence of a broad baseline inflammatory state, persistent inflammation, or impaired inflammatory resolution.

A key design consideration is that the E12.5 and E17.5 cohorts were collected at different developmental endpoints after continuous exposure from E0.5. Because the E17.5 cohort experienced 5 additional days of benzene exposure, differences between endpoints may reflect developmental age, cumulative exposure duration, or both. Microglial progenitors enter the mouse neuroepithelium early in embryogenesis and continue to mature during the later fetal period; the present study therefore examines myeloid/microglia-enriched cells after initial brain colonization has begun, rather than the initial colonization event (75–77). At the PND17 postinfection endpoint, we detected selected benzene-associated differences after MHV68 challenge. This single postnatal time point cannot determine whether those differences reflect delayed resolution, a difference in infection kinetics, or another altered response trajectory.

This two-hit model is particularly relevant for understanding how prenatal environmental exposures may contribute to later disease susceptibility (78). Many developmental exposures do not cause immediate overt abnormalities but instead shift the threshold for responding to stress, infection, or inflammation later in life (8, 79–84). In this study, MHV68 infection served as a postnatal immune challenge that revealed altered brain immune function in benzene-exposed offspring. This was most evident in males, where prenatal benzene exposure increased ORF65 levels and markedly amplified *Il1b* expression after infection. These findings suggest either impaired control of viral infection, an exaggerated inflammatory response to viral challenge, or both, although more time points will be required to fully confirm this. Importantly, this phenotype was not simply a generalized increase in all microglial markers. Instead, benzene-exposed males showed higher viral and inflammatory signals while also displaying a blunted infection-induced IBA1 response. This pattern argues for a dysregulated response rather than a uniformly hyperactivated one.

The fetal data provide a potential mechanistic foundation for this postnatal phenotype. Benzene exposure altered GPCR-linked signaling across the placenta and fetal head/brain. These pathways are biologically relevant because chemokines and lipid mediators associated with GPCR signaling are known to regulate immune cell migration, surveillance, activation, and inflammatory resolution (71). The disruption of CXCL10-CXCR3, ENPP2-LPA-LPAR3, and SPHK1-S1P-S1PR5 signaling suggests that benzene may alter how the placenta communicates inflammatory and lipid-associated signals to the fetus. However, this response was not identical across sex or developmental stage. Instead, the affected genes and directionality differed between male and female placentas and between fetal head and fetal brain. This is important because it suggests that benzene does not trigger a single uniform inflammatory pathway. Rather, it disrupts receptor-mediated signaling networks in a context-dependent manner, which may help explain why postnatal outcomes differ between males and females.

The microglial findings also support a developmental programming model. During fetal development, benzene exposure did not broadly increase IBA1 expression, suggesting that it did not simply expand or globally activate the brain myeloid compartment. Instead, benzene altered markers associated with myeloid state. At E17.5, benzene exposure reduced EGR2 and increased MHC II within CD45 ^low/int^ CD11b^+^ IBA1^+^ cells. This pattern is consistent with a shift away from a regulatory or maturation-associated phenotype and toward a more antigen-presentation-associated inflammatory state (73, 74). Because fetal microglia contribute to synaptic remodeling, vascular development, tissue surveillance, and immune education, even subtle changes in their phenotype could alter how the postnatal brain responds to infection (75–77). In this sense, the fetal microglial phenotype may represent the primed state, whereas MHV68 infection reveals the functional consequence of that priming.

Inflammatory resolution is an important mechanistic question for future study, but the present data do not establish persistent inflammation or impaired resolution. The PND17 analysis provides a single postinfection endpoint. The higher ORF65 and *Il1b* transcript abundance in benzene-exposed males could reflect differences in viral transcription, inflammatory kinetics, cell composition, or recovery trajectory. Work on persistent inflammation in perinatal brain injury provides useful conceptual context because prolonged or incompletely resolved inflammatory signaling can influence neurodevelopmental injury (85–88). However, we do not equate the current PND17 findings with persistent inflammation. Testing this hypothesis will require serial postinfection time points together with direct measures of viral kinetics, pro-resolving or repair-associated immune programs, and return toward baseline.

The sex-dependent nature of the response is an important feature of the study. Females showed benzene-associated changes in fetal morphometry, fetal transcriptional pathways, GPCR-associated signaling, and fetal myeloid markers. The lower OFD detected in benzene-exposed females at E12.5 but not E17.5 may reflect compensatory growth, recovery, or a change in growth trajectory. However, because no intermediate time points were measured, these possibilities cannot be distinguished. The E12.5 MHC II difference reflected lower MHC II in benzene-exposed males relative to benzene-exposed females, whereas at E17.5, MHC II was increased in both sexes. In the postnatal experiment, several benzene-associated differences were detected in male outcomes at PND17, whereas the female endpoints measured did not show corresponding benzene-associated differences. This pattern differs from our previous study, in which prenatal benzene exposure was associated with increased sensitivity to respiratory viral infection in females but not males (68). This suggests that the direction of sex bias after prenatal benzene exposure is context dependent and may vary by tissue, postnatal challenge, and endpoint assessed. These results support sex-dependent patterns in the endpoints evaluated, but they do not establish that males are intrinsically more susceptible to MHV68 or that females are unaffected by prenatal benzene exposure.

These findings also have broader implications for environmental health. Benzene is typically discussed as a hematotoxicant and carcinogen, but our data suggest that gestational benzene exposure can also alter fetal neuroimmune development (4). This does not mean that prenatal benzene exposure alone is sufficient to cause a specific neurodevelopmental disorder. Rather, our findings suggest that benzene exposure may alter developmental immune programming in a way that changes how the offspring brain handles later inflammatory stress. This framework may be especially relevant in real-world settings, where prenatal toxicant exposure rarely occurs in isolation and offspring are later exposed to infections, allergens, stress, or additional environmental insults (89).

The continuous 5-ppm exposure used here is a controlled experimental regimen rather than a dose-for-dose recreation of human prenatal exposure. In human populations, benzene exposure may be chronic at lower background levels and punctuated by episodic increases related to traffic, refueling, combustion, tobacco smoke, indoor sources, and occupational settings (1, 2, 12, 89–91). The present design is therefore useful for testing developmental mechanisms under sustained exposure, but it cannot define a direct human-equivalent exposure duration, intensity, or risk.

Several limitations should be considered. First, although GPCR-linked signaling emerged as a recurring pathway across placenta and fetal head/brain, this study does not prove that placental GPCR mediators directly cause the fetal microglial phenotype. Future studies using pathway blockade, targeted lipidomics, spatial transcriptomics, or placenta-specific genetic approaches will be needed to test causality. Second, the transcriptomic analyses were performed on bulk fetal head or brain tissue, which limits our ability to assign benzene-responsive genes to specific cell populations. Third, our flow cytometry approach identifies brain myeloid/microglia-enriched populations, but more detailed single-cell and spatial approaches will be needed to resolve microglial subtypes, infiltrating myeloid cells, and regional brain differences. Fourth, the postnatal infection experiment used one viral model and one postinfection time point. Because inflammatory resolution is dynamic, future studies should include a time course after infection to determine whether benzene exposure delays viral clearance, prolongs inflammatory signaling, or impairs return to baseline. Finally, this study focused on molecular and immune outcomes rather than behavioral endpoints. Future work should determine whether these neuroimmune changes are associated with altered postnatal behavior, cognition, or sensorimotor development.

Overall, this study shows that prenatal benzene exposure was associated with sex-dependent changes in fetal neuroimmune measures and selected postnatal responses to MHV68 challenge. The strongest postnatal differences were detected in males, including higher ORF65 and *Il1b* transcript abundance and altered IBA1/IL-1β responses at PND17. These observations support a developmental programming model but do not establish altered inflammatory resolution, viral clearance, or baseline brain inflammation. Serial postinfection studies are needed to determine whether prenatal exposure changes the time course of viral and inflammatory responses.

## Data Availability

The RNA sequencing data presented in this study are deposited in the NCBI Sequence Read Archive under BioProject accession number PRJNA1501637.
