# Supplementary material for: Gestational benzene exposure programs sex-specific neuroimmune responses to postnatal viral infection
Source: Front Immunol. 2026 Sep 30;17:1952071. doi: 10.3389/fimmu.2026.1952071 (PMC13661396; doi:10.3389/fimmu.2026.1952071)
Supplement: Supplementary file 1 [file DataSheet1.pdf]

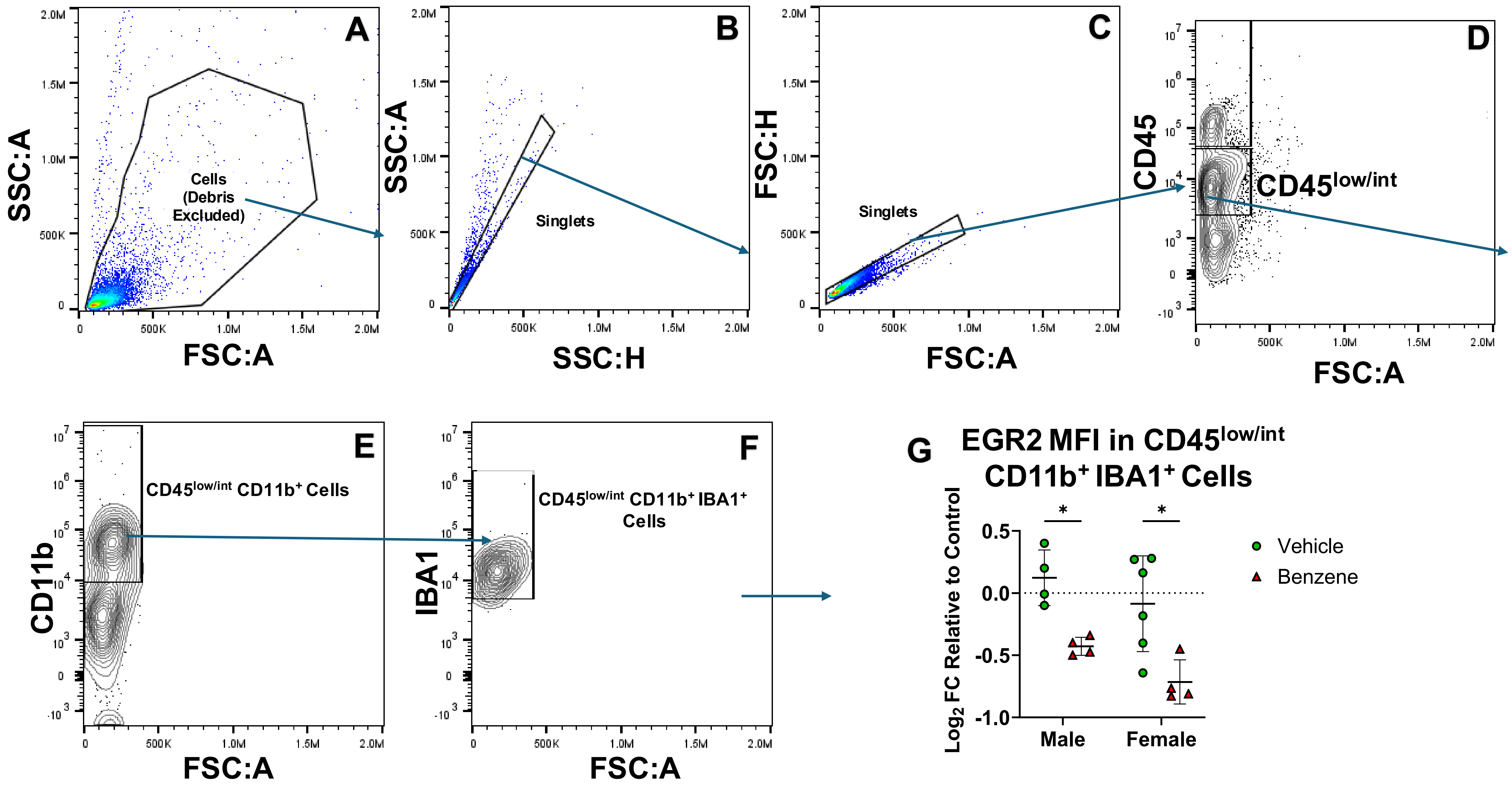
A
SSC:A
FSC:A
Cells (Debris Excluded)
B
SSC:A
SSC:H
Singlets
C
FSC:H
FSC:A
Singlets
D
CD45
FSC:A
CD45low/int
E
CD11b
FSC:A
CD45low/int CD11b+ Cells
F
IBA1
FSC:A
CD45low/int CD11b+ IBA1+ Cells
G
EGR2 MFI in CD45low/int CD11b+ IBA1+ Cells
Log2 FC Relative to Control
0.5
0.0
-0.5
-1.0
*
*
Male
Female
Vehicle
Benzene

Supplemental Figure 1. Flow cytometry gating strategy for myeloid/microglia-enriched cells.
(A) Representative FSC-A and SSC-A gating used to select cells and exclude debris from E17.5 fetal brain single-cell suspensions.
(B, C) Sequential singlet gating using SSC-A versus SSC-H and FSC-A versus FSC-H.
(D) Identification of $CD45^{low/int}$ cells.
(E) Gating of $CD45^{low/int}$ CD11b+ fetal brain myeloid cells.
(F) Gating of $CD45^{low/int}$ $CD11b^{+}$ $IBA1^{+}$ fetal brain myeloid/microglia-enriched cells.
(G) Representative quantification of EGR2 MFI in $CD45^{low/int}$ $CD11b^{+}$ $IBA1^{+}$ cells, shown as $\log_2$ fold change relative to sex-matched vehicle controls.
Gates were established using single-color controls prepared with cells and compensation beads, with gate placement guided by positive staining patterns and background fluorescence across channels. Gates were applied consistently across all samples.

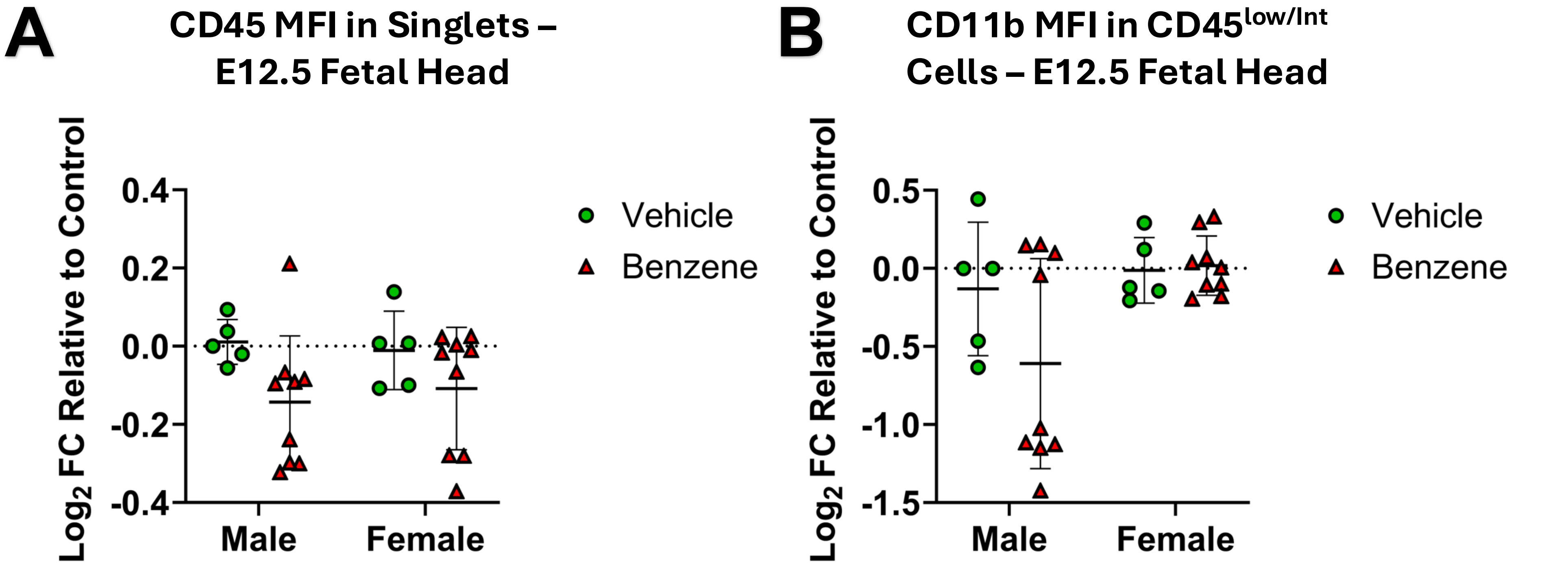

A
CD45 MFI in Singlets – E12.5 Fetal Head
Log2 FC Relative to Control
0.4
0.2
0.0
-0.2
-0.4
Male
Female
Vehicle
Benzene
B
CD11b MFI in CD45low/Int Cells – E12.5 Fetal Head
Log2 FC Relative to Control
0.5
0.0
-0.5
-1.0
-1.5
Male
Female
Vehicle
Benzene

Supplemental Figure 2. Gestational benzene exposure does not consistently alter CD45 or CD11b MFI in E12.5 fetal head.
(A) CD45 MFI in debris-excluded singlets from E12.5 fetal head, stratified by fetal sex and exposure group.
(B) CD11b MFI in $CD45^{low/int}$ cells from E12.5 fetal head, stratified by fetal sex and exposure group.
Each data point represents an individual fetus. Fetuses were collected from three independent dams/litters, with samples distributed across litters. Data are presented as mean ± SD. Statistical comparisons were performed using two-way ANOVA with Tukey's post hoc test where appropriate. $P < 0.05$ (*), $P < 0.01$ (**), $P < 0.001$ (***), $P < 0.0001$ (****).

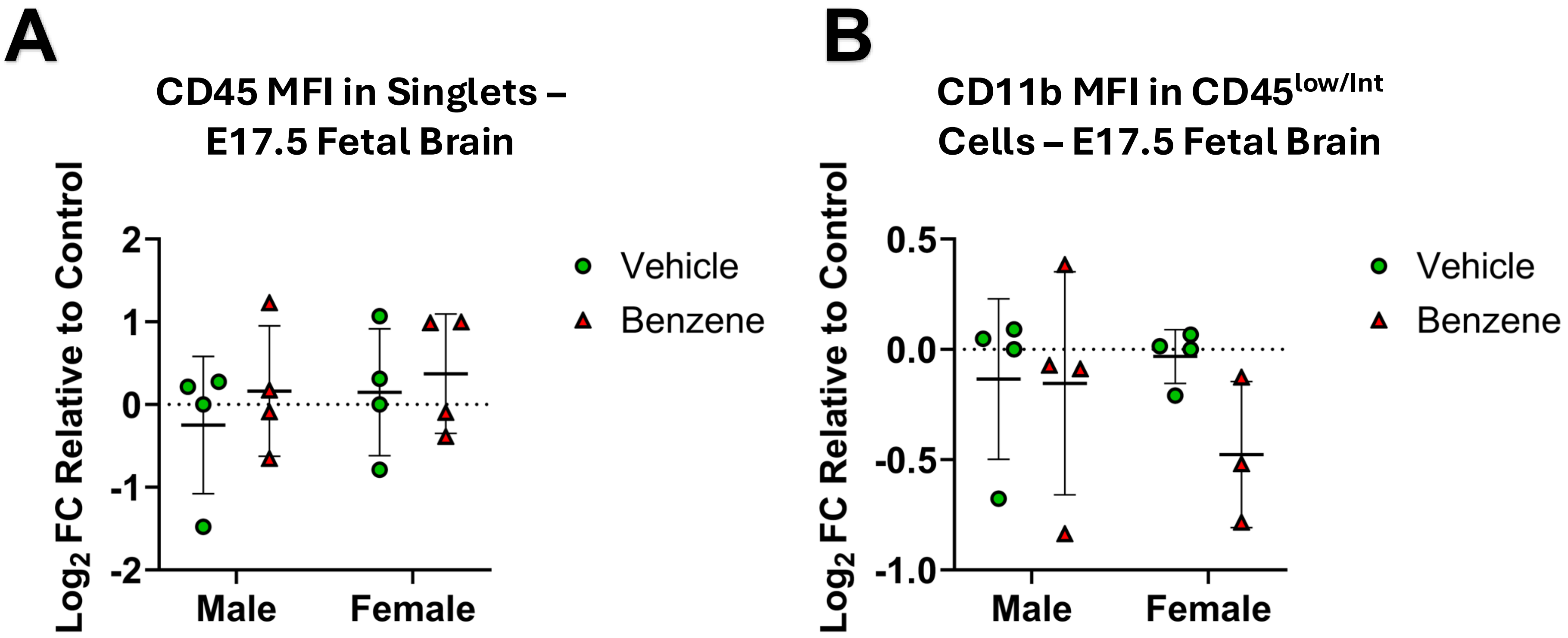
A
CD45 MFI in Singlets –
E17.5 Fetal Brain
Log2 FC Relative to Control
2
1
0
-1
-2
Male
Female
Vehicle
Benzene
B
CD11b MFI in CD45low/Int
Cells – E17.5 Fetal Brain
Log2 FC Relative to Control
0.5
0.0
-0.5
-1.0
Male
Female
Vehicle
Benzene

Supplemental Figure 3. Gestational benzene exposure does not consistently alter CD45 or CD11b MFI in E17.5 fetal brain.
(A) CD45 MFI in debris-excluded singlets from E17.5 fetal brain, stratified by fetal sex and exposure group.
(B) CD11b MFI in $CD45^{low/int}$ cells from E17.5 fetal brain, stratified by fetal sex and exposure group.
Each data point represents an individual fetus. Fetuses were collected from three independent dams/litters, with samples distributed across litters. Data are presented as mean ± SD. Statistical comparisons were performed using two-way ANOVA with Tukey's post hoc test where appropriate. P < 0.05 (*), P < 0.01 (**), P < 0.001 (***), P < 0.0001 (****).

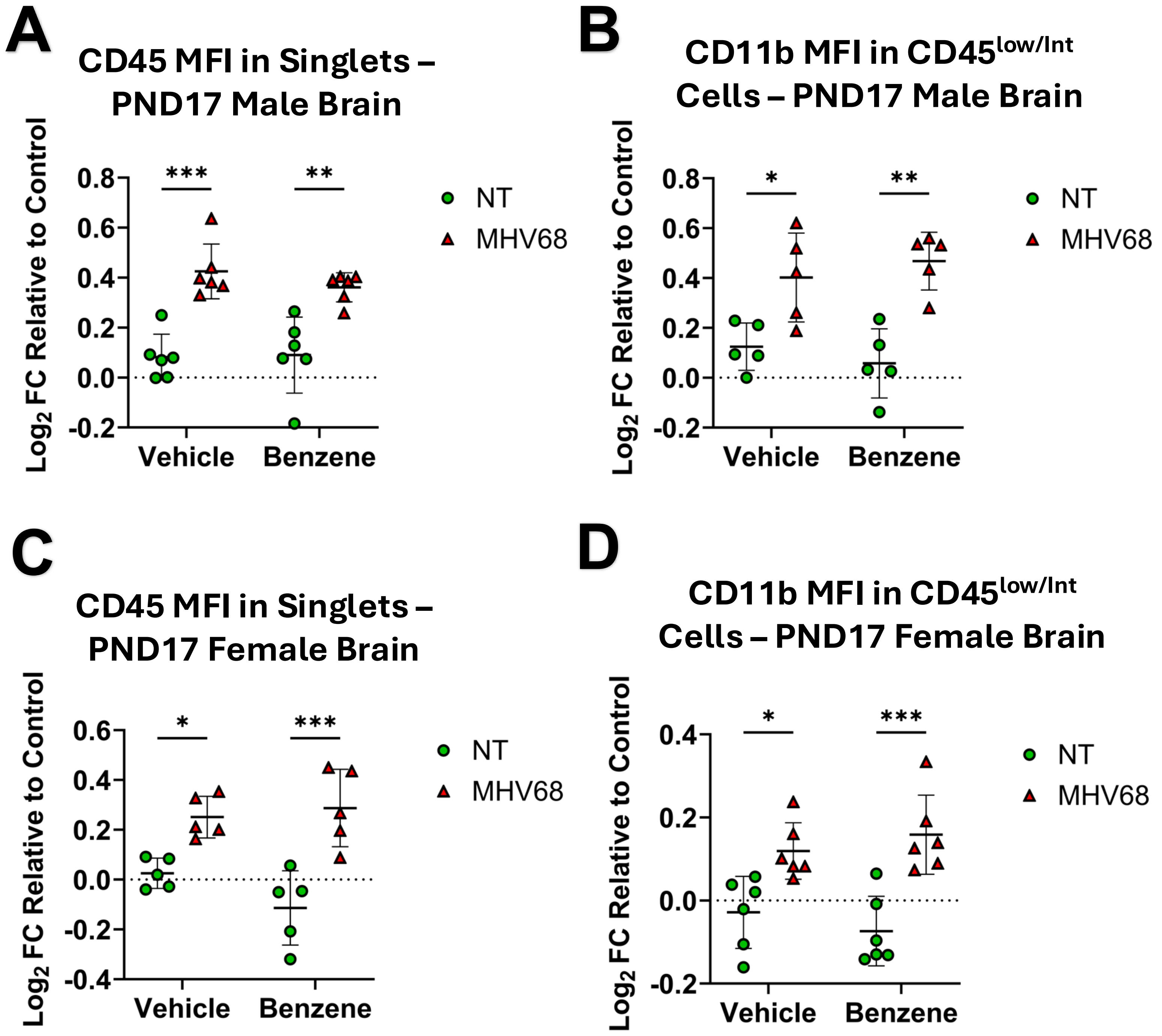

A
CD45 MFI in Singlets – PND17 Male Brain
Log2 FC Relative to Control
0.8
0.6
0.4
0.2
0.0
-0.2
***
**
Vehicle
Benzene
NT
MHV68
B
CD11b MFI in CD45low/Int Cells – PND17 Male Brain
Log2 FC Relative to Control
0.8
0.6
0.4
0.2
0.0
-0.2
*
**
Vehicle
Benzene
NT
MHV68
C
CD45 MFI in Singlets – PND17 Female Brain
Log2 FC Relative to Control
0.6
0.4
0.2
0.0
-0.2
-0.4
*
***
Vehicle
Benzene
NT
MHV68
D
CD11b MFI in CD45low/Int Cells – PND17 Female Brain
Log2 FC Relative to Control
0.4
0.2
0.0
-0.2
*
***
Vehicle
Benzene
NT
MHV68

Supplemental Figure 4. Prenatal benzene exposure and postnatal MHV68 infection alter CD45 and CD11b MFI in PND17 offspring brain.

(A) CD45 MFI in debris-excluded singlets from PND17 male offspring brain following prenatal vehicle or benzene exposure and postnatal uninfected or MHV68 challenge.

(B) CD11b MFI in $CD45^{low/int}$ cells from PND17 male offspring brain following prenatal vehicle or benzene exposure and postnatal uninfected or MHV68 challenge.

(C) CD45 MFI in debris-excluded singlets from PND17 female offspring brain following prenatal vehicle or benzene exposure and postnatal uninfected or MHV68 challenge.

(D) CD11b MFI in $CD45^{low/int}$ cells from PND17 female offspring brain following prenatal vehicle or benzene exposure and postnatal uninfected or MHV68 challenge.

Each data point represents an individual offspring. Offspring were collected from three independent dams/litters, with samples distributed across litters. Data are presented as mean ± SD. Statistical comparisons were performed using two-way ANOVA with Tukey's post hoc test where appropriate. $P < 0.05$ (*), $P < 0.01$ (**), $P < 0.001$ (***), $P < 0.0001$ (****).
